# Characterization of a *Trichinella spiralis* aminopeptidase and its participation in invasion, development and fecundity

**DOI:** 10.1186/s13567-020-00805-w

**Published:** 2020-06-15

**Authors:** Kai Xia Guo, Ying Bai, Hua Nan Ren, Xiang Yuan Sun, Yan Yan Song, Ruo Dan Liu, Shao Rong Long, Xi Zhang, Peng Jiang, Zhong Quan Wang, Jing Cui

**Affiliations:** Department of Parasitology, Medical College, Zhengzhou University, Zhengzhou, 450052 China

## Abstract

A *Trichinella spiralis* aminopeptidase (TsAP) has been identified in intestinal infectious larvae (IIL) and adult worms (AW), but its biological function in the *T. spiralis* life cycle is unknown. The aim of this study was to characterize TsAP and ascertain its functions in the invasion, development and fecundity of *T. spiralis*. Recombinant TsAP (rTsAP) was expressed and purified. rTsAP has strong immunogenicity. qPCR and western blotting show that TsAP was transcribed and expressed at all *T. spiralis* lifecycle stages, but the expression level of TsAP mRNA and proteins at IIL and AW stages was obviously higher than those in muscle larvae (ML) and newborn larvae (NBL). The IFT results reveal that TsAP was principally located at the cuticle and the intrauterine embryos of this nematode. rTsAP had the enzymatic activity of natural aminopeptidase to hydrolyze the substrate Leu-pNA with an optimal temperature of 50 °C and optimal pH of 8.0. rTsAP promoted the larval penetration into intestinal epithelial cells, whereas anti-rTsAP antibodies suppressed the larval intrusion; the promotion and suppression was dose-dependently related to rTsAP or anti-rTsAP antibodies. TsAP protein expression level and enzymatic activity were reduced by 50.90 and 49.72% through silencing of the TsAP gene by specific siRNA 842. Intestinal AW and muscle larval burdens, worm length and female reproductive capacity were significantly declined in mice infected with siRNA-transfected ML compared to the control siRNA and PBS group. These results indicate that TsAP participates in the invasion, development and fecundity of *T. spiralis* and it might be a candidate target for anti-*Trichinella* vaccines.

## Introduction

*Trichinella spiralis* is an enzootic tissue-parasitizing nematode that infects more than 150 kinds of mammalian animals in the world [1]. *Trichinella* infection in humans primarily results from the ingestion of infective muscle larvae (ML) contained in raw or undercooked animal meat. In mainland China, 12 human trichinellosis outbreaks owing to infected pork or pork products occurred from 2004 to 2009 [2]. Pork and pork products of domestic pigs still are the major infectious source of human *Trichinella* infection in developing countries [3, 4]. *Trichinella* infection is not only an important public health problem but also a tremendous threat to meat food safety [5, 6].

When contaminated meat is ingested, the encapsulated *T. spiralis* ML in muscles are first liberated in the host’s stomach and then activated to intestinal infectious larvae (IIL) by enteral contents or bile [7]. The IIL larvae penetrate into the intestinal epithelium and mature into adulthood after four molts. The female adult worms (AW) deposit the newborn larvae (NBL), which enter the blood system and intrude the skeletal muscle and develop into the ML stage, and the lifecycle is completed [8, 9]. The intestinal mucosa is the first native protective screen against *Trichinella* infection and the preferential interaction location between this nematode and the host [10]. However, the mechanism of *Trichinella* intrusion into the intestinal epithelium has not yet been fully elucidated. The characterization of intrusion-related *Trichinella* proteins will be valuable to understand the interaction mechanism of *T. spiralis* with the intestinal epithelium and develop vaccines against *Trichinella* invasive stage worms [11].

Previous studies showed that when they were inoculated onto intestinal epithelial cell (IEC) monolayer, *T. spiralis* IIL larvae intrude the monolayer and generate several proteases, and some of these proteases passed into the IEC [12, 13]. A *T. spiralis* aminopeptidase (TsAP, GenBank: XP_003377703.1) was identified amongst the proteases produced by the IIL larvae after co-cultivation with IEC. Additionally, TsAP was also identified in AW crude proteins and excretion/secretion (ES) recognized by anti-*Trichinella* antibodies from infected pigs and trichinellosis patients [14, 15]. Aminopeptidase is a collection of peptidases that catalyze the hydrolysis of residues from the amino terminus of peptides and proteins. They might play important physiological functions, such as degradation of the host’s peptides and proteins, modulation of gene expression, antigen processing and defense [16]. In a previous study, TsAP was cloned into the GEX-6p-1, but the rTsAP was expressed in the form of inclusion bodies, and immunization of mice with rTsAP exhibited an obvious immune protection against *T. spiralis* larval challenge [17]. TsAP might be involved in *T. spiralis* intrusion of the host enteral mucosa and it likely is a potential target for anti-*Trichinella* vaccine, but its biological roles in the *T. spiralis* life cycle has not been identified up to now.

The aim of the present work was to investigate the physiological and biochemical characteristics of TsAP, and to ascertain its functions in worm invasion, development and fecundity of *T. spiralis*.

## Materials and methods

### Parasite and experimental animals

*Trichinella spiralis* isolate (ISS534) used in this study was collected from a naturally infected domestic pig in central China [18]. Six-week-old female BALB/c mice were obtained from the Henan Provincial Experimental Animal Center (Zhengzhou, China).

### Worm collection and soluble protein preparation

*Trichinella spiralis*-infected murine muscles at 42 days post-infection (dpi) were digested by an artificial digestion method to collect the ML [19]. IIL were harvested from infected murine small intestines at 6 h post-infection (hpi) [20]. AW were obtained from murine intestine at 3 dpi [21]. Female adults were cultured in RPMI-1640 at 37 °C in 5% CO_2_ for 24 h, the NBL were harvested [22]. The soluble worm somatic proteins of ML, IIL, AW and NBL were prepared as previously established [23].

### Bioinformatics analysis of TsAP

Full-length cDNA sequence of the TsAP gene was retrieved from GenBank (Accession no: XP_003377703.1). The characteristics of the TsAP gene were ascertained through bioanalysis software and websites. The tertiary structure of TsAP protein was predicted by PyMOL, and its functional sites were analyzed using CN3D [10]. The amino acid sequence of TsAP was compared with aminopeptidases from other organisms using Clustal X [24]. The GenBank accession numbers of aminopeptidase from other organisms were as follows: *Trichinella murrelli* (KRX40782), *T. britovi* (KRY54954), *Trichinella* sp. T6 (KRX83552), *T. patagoniensis* (KRY23873), *T. nativa* (KRZ63104), *Trichinella* sp. T8 (KRZ88184), *T. nelsoni* (KRX23279), *T. pseudospiralis* (KRX87671), *T. zimbabwensis* (KRZ07724), *T. papuae* (KRZ79909), *Trichuris trichiura* (CDW53285), *Clonorchis sinensis* (RJW66865), *Toxoplasma gondii* (KFG62794.1), *Plasmodium vinckeipetteri* (EUD71337), *Echinococcus granulosus* (CDS20652), *Brugia malayi* (CTP81255), *Caenorhabditis elegans* (NP_498854). Two sequences of *Homo sapiens* (AAD17527) and *Mus musculus* (NP_077754) were used as the outgroups. The phylogenetic analysis was performed in MEGA 7.0 based on the Neighbour-joining (NJ) method as described previously [25].

### Cloning and expression of recombinant TsAP (rTsAP)

Total RNA were isolated from the ML using Trizol (Invitrogen, USA). The full-length TsAP cDNA sequence was amplified by PCR using specific primers carrying restriction enzyme sites BamHI and HindIII **(bold**) (5′-ATA**GGATCC**ATGAGCCGCAAAGGATTGATG-3′,5′ CCC**AAGCTT**TCAACTAGATTTTGCCAAAAG-3′). The PCR products were cloned into the expression vector pQE-80L (Novagen, USA), then the recombinant pQE-80L/TsAP was transformed into *Escherichia coli* BL21 (Novagen, USA). The rTsAP expression was induced using 0.8 mM IPTG for 24 h at 16 °C [26], subsequently purified using Ni–NTA-Sefinose resin (Sangon Biotech Co., Shanghai, China) [27]. The concentration of rTsAP protein was determined by Coomassie brilliant blue method and analyzed by SDS-PAGE as reported before [28].

### Immunization of mice and assay of polyclonal anti-rTsAP antibodies

Twenty mice were subcutaneously injected with 20 μg rTsAP emulsified with complete Freund’ adjuvant. Three boost immunizations were administered by 20 μg rTsAP emulsified with incomplete Freund’ adjuvant at a 2-week-interval [29, 30]. At 1 week following the final immunization, tail blood was collected and anti-rTsAP sera were isolated [31].

Serum levels of anti-rTsAP antibody IgG in all vaccinated mice were determined by ELISA with rTsAP [32]. Briefly, the ELISA plate was coated with 2 μg/mL rTsAP at 4 °C overnight. After washing with PBST, the plate was blocked with 5% skimmed milk at 37 °C for 2 h. Serial dilutions of mouse immune sera were incubated at 37 °C for 1 h, followed by the incubation of 1:10,000 dilutions of HRP-conjugated anti-mouse IgG for 1 h at 37 °C. Plates were developed with substrate o-phenylenediamine dihydrochloride (OPD; Sigma) plus H_2_O_2_, the reaction was terminated by addition of 2 M H_2_SO_4_. The absorbance at 492 nm was assayed by a microplate reader (Tecan, Schweiz, Switzerland) [24, 33].

### Western blot analysis of antigenicity and expression level of TsAP protein

Soluble somatic proteins of various *T. spiralis* stages (ML, IIL, 3 dpi AW and NBL) and rTsAP were identified by western blotting with anti-rTsAP serum. Worm proteins and rTsAP were separated by SDS-PAGE, then transferred onto nitrocellulose membrane (Merck Millipore, MA, USA) at 18 V for 35 min [34]. The membrane was cut into strips that were blocked using 5% skimmed milk in TBST at 37 °C for 2 h. Following washing with TBST, the strips were reacted with anti-rTsAP serum (1:100) for 1 h at 37 °C, and followed by incubation of HRP-conjugated anti-mouse IgG (1:10,000; Sigma-Aldrich, USA) at 37 °C for 1 h. After washing again, the strips were developed with 3, 3′-diaminobenzidine tetrahydrochloride (DAB; Sigma-Aldrich), and finished by washing the membrane with deionized water [21, 35].

To ascertain the relative TsAP protein expression level at various *T. spiralis* stages, 15 μg/lane of soluble proteins of ML, IIL, 3 dpi AW and NBL was analyzed using SDS-PAGE and western blot with 1:100 dilutions of anti-rTsAP serum [36]. An antibody against GAPDH (1:1000) was used to assess GAPDH expression as an internal quantitative control [37]. After washing, the strip was colored with an enhanced chemiluminescent kit (CWBIO, Beijing, China) [29]. The relative TsAP protein expression levels in *T. spiralis* different stages were analyzed by Image J software.

### qPCR analysis of TsAP transcription level

Total RNA from various *T. spiralis* stages (ML, IIL, 3 dpi AW and NBL) were isolated using Trizon reagent (Invitrogen, USA), reverse-transcribed into cDNA using PrimeScript™ RT reagent Kit (TaKaRa, Japan). The transcriptional level of TsAP at various worm stages was assessed using qPCR as reported before [38, 39]. The specific primers of qPCR for amplifying the TsAP gene were 5′-TCG CAA CTT TGA CTG GAG CA-3′, and 5′-GGA AGA CGC CAA ACA CGT TC-3′. The TsAP transcription level was normalized by subtracting the transcription level of a *T. spiralis* housekeeping gene GAPDH (GenBank: AF452239), and then calculated on the basis of a comparative Ct (2^−ΔΔCt^) method [11]. Each sample had three replicates.

### Immunofluorescence test (IFT)

The recognition of natural TsAP at different *T. spiralis* stages (ML, IIL and AW) was examined by IFT with the worm cross-sections as reported before [40, 41]. The worms were embedded in paraffin, 2-µm thick worm cross-sections were prepared and blocked with 5% normal goat serum at 37 °C for 1 h. After washing with PBS, the sections were incubated with 1:10 dilution of various sera (anti-rTsAP serum, infection serum and normal serum) at 37 °C for 1 h. After washing again with PBS, the sections were stained at 37 °C for 1 h by FITC- conjugated anti-mouse IgG (1:100; Santa Cruz, USA) and observed under fluorescent microscopy (Olympus, Japan) [42, 43].

### Enzymatic activity assay of rTsAP

To determine the enzymatic activity of rTsAP, the serially diluted rTsAP (0, 0.004, 0.008, 0.016, 0.032, 0.064 and 0.128 μg/μL) was pre-incubated at 37 °C for 10 min in various pH buffers (pH 4.0–5.0 sodium acetate, pH 6.0–7.0 sodium phosphate, pH 8.0–9.0 Tris–HCl, and pH 10.0–11 sodium bicarbonate) [44]. Subsequently, the aminopeptidase substrate 1 mM Leu-P-nitroaniline (Leu-pNA, Sigma-Aldrich, USA) was added into the reaction mixture and incubated at different temperatures (20–100 °C) for 15 min [45], and the absorbance at 405 nm was measured with a spectrophotometer. In order to verify whether the rTsAP enzymatic activity is metal ion-dependent, four common auxiliary metal ions (Co^2+^, Zn^2+^, Mn^2+^ and Ni^2+^) were selected and added into the reaction system at the same concentration (0.5 mM) to analyze their ability to affect rTsAP activity [46]. Different enzymatic inhibitors (2 mM 1, 10-Phenanthroline, 1 mM AEBSF and 5 μM E-64) were used to determine the effects of various inhibitors on the rTsAP enzyme activity [45]. The reaction rates of rTsAP to hydrolyze the substrate Leu-pNA at different concentrations (0.5, 1, 2, 3, 4, 5, 6, 7 and 8 mM) were calculated according to the product standard curve, and the Michaelis–Menten plot and Lineweaver–Burk plot were performed to obtain the K_m_ value [39, 47]. The effects of temperature, pH, metal ions and enzymatic inhibitors on rTsAP activity were calculated as the mean and SD of three independent tests.

### Binding of rTsAP and IEC proteins determined by ELISA

Primary IEC were separated from mouse intestine and susceptive to *Trichinella* invasion [8]. Mouse striated muscle myoblast C2C12 was non-susceptive to *Trichinella* invasion and utilized as negative control [48, 49]. The cells were cultivated in Dulbecco modified Eagle media and harvested using trypsinization. The soluble proteins of IEC and C2C12 were prepared by grinding, sonication and centrifugation, and their protein concentration was assessed as reported before [39].

The binding between rTsAP and IEC proteins was ascertained by ELISA [50, 51]. Briefly, the microplates were coated with serial diluted IEC proteins (0.01, 0.02, 0.04, 0.08, 0.16, 0.32 and 0.64 μg/mL) overnight at 4 °C. After blocking with 5% skimmed milk and washing with PBST, the plates were incubated with serial diluted rTsAP proteins (0.10, 0.25, 0.50, 1.00, 2.00, 4.00 and 8.00 μg/mL) at 37 °C for 2 h. After washing again, the plates were probed with 1:100 dilution of diverse murine sera (anti-rTsAP serum, infection serum or normal serum), followed by the incubation of HRP-conjugated anti-mouse IgG (1:10,000, Sigma). Coloration was developed with OPD (Sigma), and absorbance at 492 nm was determined [52]. All samples were performed in duplicate.

### Binding of rTsAP and IEC proteins determined by Far Western blotting

The binding of rTsAP and IEC was examined by Far-western blotting. In brief, the IEC lysates were separated by SDS-PAGE with 12% separation gel as described [15]. The proteins were transferred onto nitrocellulose membrane which was cut into strips, blocked, and probed with anti-rTsAP serum. After washing with PBST, the strips were incubated with HRP-conjugated anti-mouse IgG (1:10,000, Sigma, USA). After washing, coloration was performed with DAB (Sigma) [53].

### Binding of rTsAP and IEC assessed by IFT and confocal microscopy

The binding of rTsAP and IEC and its cellular location was also investigated using IFT and confocal microscopy [39]. The IEC were cultured on coverslip in a 6-well plate in DMEM medium [54]. When the IEC cells were grown to confluence, the IEC monolayer was pre-incubated with 20 µg/mL rTsAP at 37 °C for 2 h, and then blocked with 5% goat serum for 1 h. Following washing with PBS, the monolayer was probed using 1:10 dilutions of anti-rTsAP serum at 37 °C for 1 h. After washing, the cells were dyed at 37 °C for 1 h using FITC-conjugated anti-mouse IgG (1:100, Santa Cruz, USA), the cell nuclei were re-dyed using propidium iodide (PI) for 5 min and examined under a fluorescent microscope (Olympus, Japan). Eventually, the cellular localization of TsAP within the IEC was observed under confocal microscopy [55].

### The in vitro larval penetration into IEC

To evaluate the TsAP role in the process of larval penetration into intestinal epithelium, the in vitro penetration test was conducted as previously described [48, 56]. Briefly, the ML were first co-incubated with 5% swine bile at 37 °C for 2 h, which activated the ML into the IIL larvae. One hundred IIL larvae were added onto IEC monolayer cultured in semisolid medium [8]. The medium was pre-supplemented with serial diluted rTsAP protein (0, 4, 8, 12, 16, 20 and 24 μg/mL) or serial dilutions (1:100–1:1200) of anti-rTsAP serum, infection serum or pre-immune serum. Additionally, BSA and the specific enzyme inhibitor (1, 10-Phenanthroline) were used as the control. After incubation at 37 °C for 2 h, larval penetration into the monolayer was examined under a microscope. The parasites penetrating into the monolayer and migrating into it were assessed as penetrating parasites, whereas the worms still existing on the surface of the cell monolayer and exhibiting a spiral coil were assessed as the non-penetrated worms [40]. Each sample has triplicates.

### RNA interference

The TsAP-specific siRNA 842 (5′-GGAUCUAUGCGUUUCGAUATT-3′) was designed according to the full-length TsAP cDNA sequence and prepared by Sangon Biotech Co., Ltd (Shanghai, China). A control siRNA with a scrambled sequence (5′-AUCGGCUACCAAG UCAUACTT-3′) was also prepared [57]. *Trichinella spiralis* ML were transfected using siRNA 842 by electroporation, and then cultivated at 37 °C for 7 days in RPM1640 medium. The crude ML somatic proteins were prepared. TsAP protein expression level of siRNA-transfected and control ML was ascertained on Western blotting analysis, and GAPDH protein expression level was also assessed as a housekeeping gene control [37]. Enzyme activity of native TsAP in soluble proteins of siRNA 842 treated ML was investigated and compared to those of non-treated ML by the abovementioned method. Suppression of siRNA 842 on worm intrusion, development and fecundity was investigated as previously reported [58].

### Challenge of mice with siRNA 842-transfected ML

In order to ascertain the in vivo invasive and developmental capacity of siRNA 842-transfected ML, sixty mice were divided equally into 3 groups, and each mouse was infected orally with 300 ML treated with 3 μM siRNA 842, control siRNA and PBS, respectively. Ten mice of each group were killed to harvest the AW from small intestines at 6 dpi [59]. An additional 10 mice from each group were euthanized at 60 dpi, and the ML were recovered by artificial digestion of mouse carcasses [19]. The worm reduction was assessed on the basis of the number of enteral AW and muscle larvae per gram (LPG) of tissues recovered from siRNA 842 group compared to those from only the PBS group [60]. Thirty females from each group were cultured, and the reproductive capacity of female AW was ascertained in line with the NBL produced by each female in 72 h. The morphology of AW, NBL and ML from each group were examined under a microscope, their images were acquired, and the worm length was measured with an imaging software measuring tool (CellSens version 1.5) [61].

### Statistical analysis

All data were analyzed with SPSS 20.0 software, and the results are presented as the mean ± standard deviation (SD). One-way ANOVA was used to analyze the difference in the relative TsAP expression levels, worm burdens, NBL production and lengths among the groups. A Chi square test was used to compare the differences of worm invasion rate among various groups. The correlation between the rTsAP dose and worm invasion was analyzed by linear regression. *P* < 0.05 was defined as statistical significance.

## Results

### Bioinformatics analysis of TsAP

The full-length cDNA sequence of TsAP gene is 1515 bp encoding 504 amino acids, with a 54.7 kDa molecular weight (MW) and 6.69 isoelectric point (pI). TsAP had no signal peptide site. The homology comparison of TsAP amino acid sequences with aminopeptidase of other species or genotypes of the genus *Trichinella* are shown in Additional file 1. The amino acid sequences of the TsAP have an identity of 99.40, 99.21, 98.61, and 98.61% with aminopeptidases of the 4 encapsulated *Trichinella* species (*T. murrelli*, *T. britovi*, *T. nativa* and *T. nelsoni*), and it has an identity of 97.62, 97.22 and 97.22% of those from 3 non-encapsulated *Trichinella* species (*T. pseudospiralis*, *T. zimbabwensis* and *T. papuae*).

**Figure 1:**
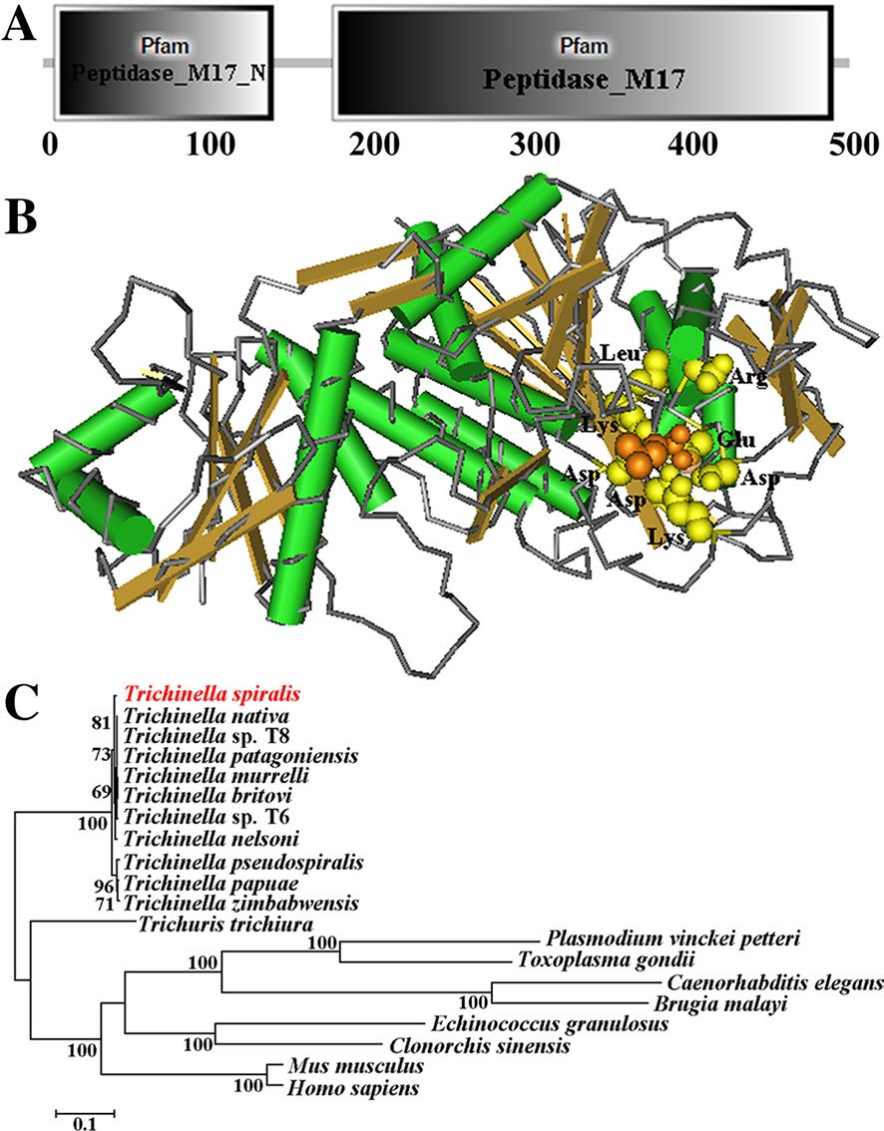
**The predicted 3-dimensional structure of TsAP protein (A, B) and phylogenetic trees of aminopeptidases of 20 organisms with the NJ method (C). A** Predicted two domains of TsAP (Peptidase_M17_N and Peptidase_M17). **B** Predicted eight enzymatic activity sites (Lys, Asp, Lys, Asp, Asp, Glu, Arg and Leu), which are signed as yellow.

The SMART analysis showed that TsAP contained two domains of Peptidase_M17 (a Peptidase_M17_N and another Peptidase_M17 domain), which has a catalysis function for the hydrolysis of N-terminal amino acid residues (Figure 1A). In the three-dimensional model, TsAP has eight enzymatic activity sites located at Lys264, Asp269, Lys276, Asp287, Asp348, Glu350, Arg352 and Leu376, respectively (Figure 1B). Phylogenetic analysis of TsAP with an aminopeptidase from other helminths are shown in Figure 1C. As shown in the tree, the monophyletic group of the genus *Trichinella* was well supported, which has a close relationship with the intestinal parasitic nematode *Trichuris trichiura*. Within the genus *Trichinella*, two distinct clades were revealed: one was the encapsulated clade (including *T. spiralis*, *T. nelsoni*, T6, *T. murrelli*, *T. britovi*, *T. patagoniensis*, *T. nativa* and T8), the other was the non-encapsulated clade (*T. pseudospiralis*, *T. zimbabwensis* and *T. papuae*).

### Anti-rTsAP antibody IgG response to rTsAP immunization

The mice were immunized using rTsAP for four times. Serum anti-rTsAP antibody IgG titers at 1 week after the final immunization were tested by ELISA. Anti-rTsAP IgG levels in immunized mice achieved to 1:100,000 after the last immunization, demonstrating that rTsAP has strong immunogenicity.

### Expression and identification of rTsAP

**Figure 2:**
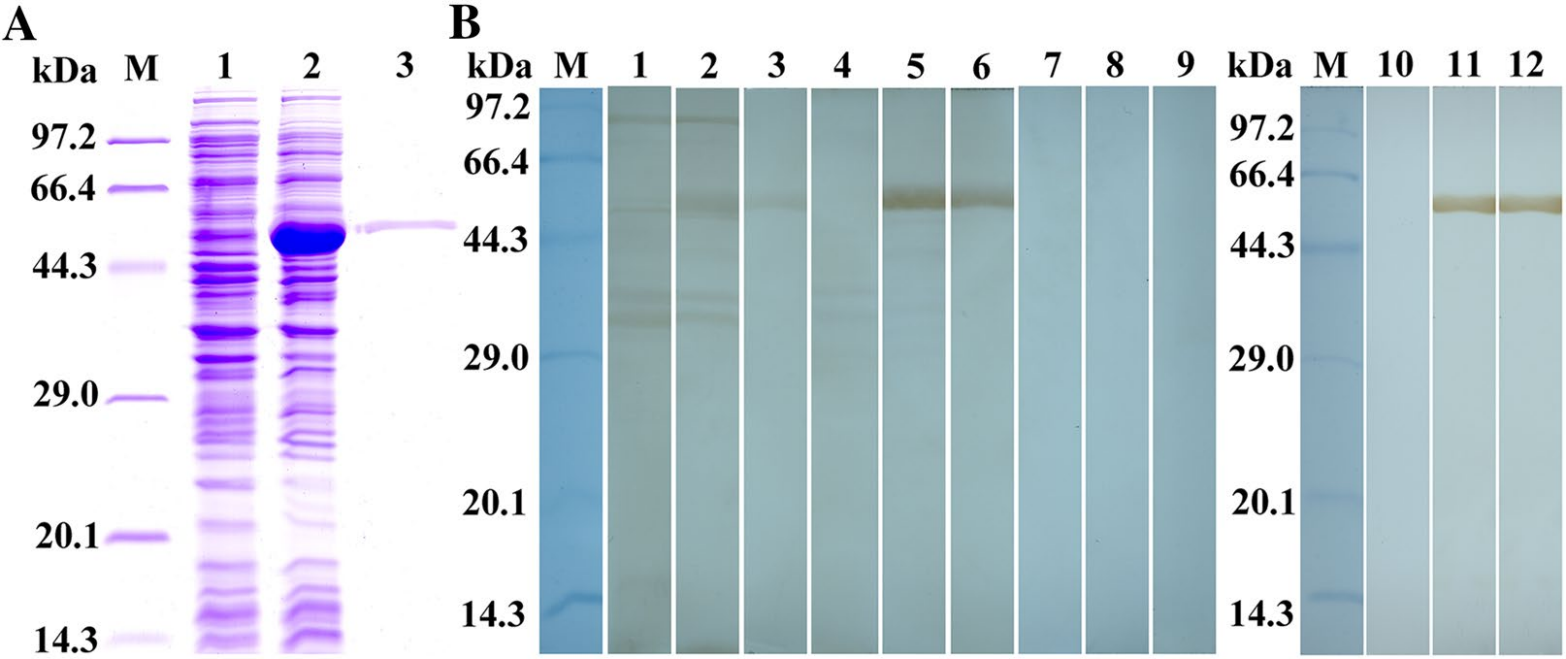
**Identification of the rTsAP. A** SDS-PAGE analysis of the rTsAP. Lane M: Protein marker; Lane 1: lysate of recombinant *E. coli* incorporating pQE-80L/TsAP prior to induction; Lane 2: lysate of recombinant *E. coli* incorporating pQE-80L/TsAP following induction; Lane 3: the purified rTsAP. **B** Western blot analysis of the rTsAP. The lysates of pQE-80L/TsAP prior to induction (lanes 1, 4, 7 and 10) were not recognized by infection serum (lane 1), anti-rTsAP serum (lane 4), normal serum (lane 7) and anti-his McAb (lane 10). The lysates of pQE-80L/TsAP following induction (lanes 2, 5, 8 and 11) and rTsAP (lanes 3, 6, 9 and 12) were probed by infection serum (lanes 2 and 3), anti-rTsAP serum (lanes 5 and 6) and anti-his McAb (lanes 11 and 12), but not by normal serum (Lanes 8 and 9).

The results of SDS-PAGE analysis revealed that the BL21 bacteria carrying pQE-80L/TsAP expressed a band of 55.7 kDa fusion protein. After purification with Ni–NTA Sefinose Column, the rTsAP protein showed a clear individual band (Figure 2A). The molecular weight (55.7 kDa) of rTsAP was consistent with its predicted size. By western blot analysis, the purified rTsAP was identified by anti-rTsAP serum, infection serum and anti-his tag monoclonal antibody (McAb), but not by normal murine serum (Figure 2B).

### Western blot analysis of TsAP protein expression in diverse stages

**Figure 3:**
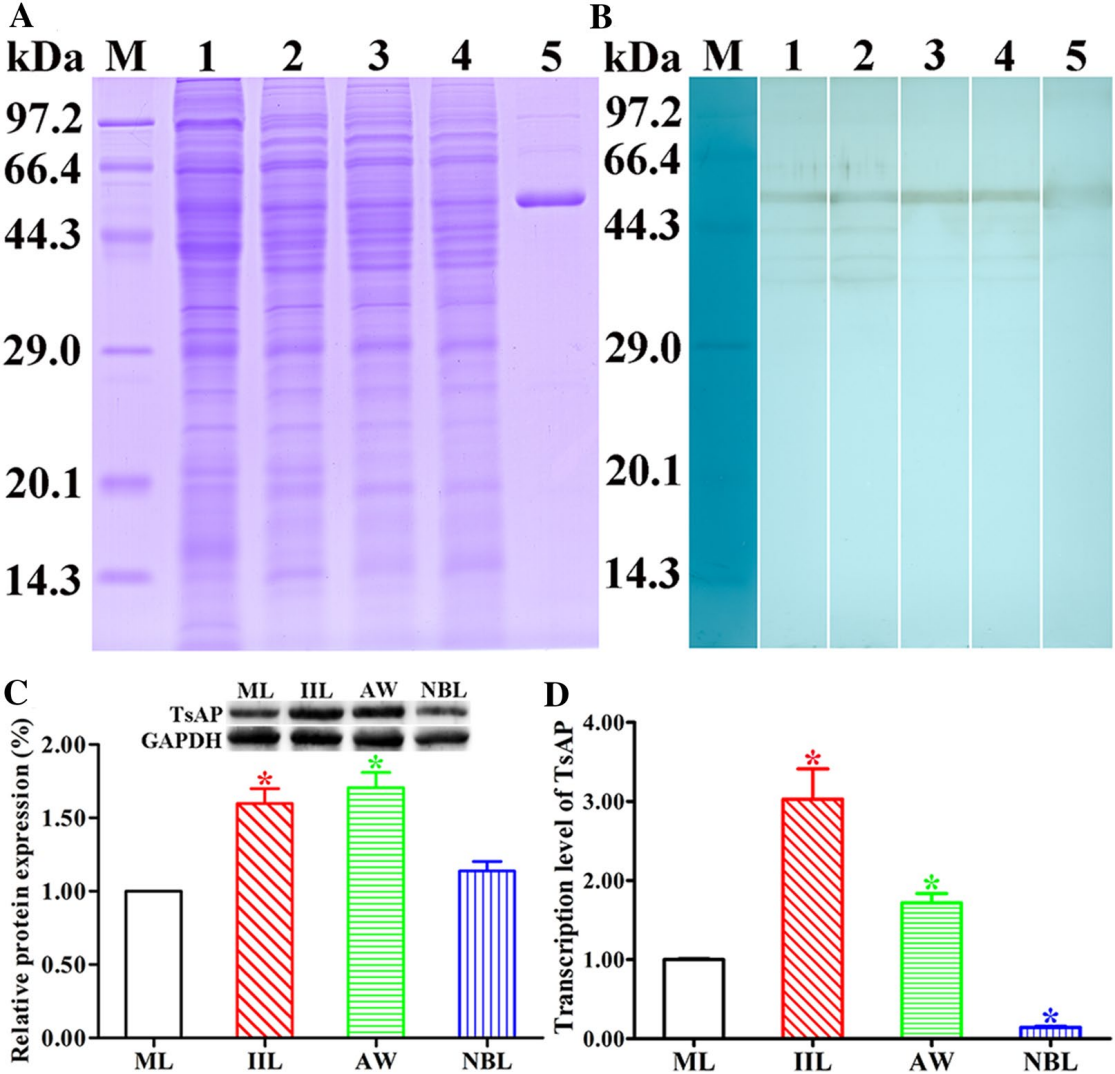
**Western blot and qPCR analysis of TsAP protein and mRNA expression levels at diverse*T. spiralis*stages. A** SDS-PAGE analysis of crude proteins of diverse *T. spiralis* stages. Lane M: protein markers; Lane 1: ML; Lane 2: IIL; Lane 3: AW; Lane 4: NBL; Lane 5: rTsAP. **B** Identification of native TsAP in crude proteins of *T. spiralis* ML (lane 1), IIL (lane 2), AW (lane 3) and NBL (lane 4), and rTsAP (lane 5) by western blot analysis with anti-rTsAP serum. **C** Quantitative analysis of TsAP expression levels in crude proteins of diverse *T. spiralis* stages (ML, IIL, AW and NBL) was determined by western blot with 1:100 dilutions of anti-rTsAP serum. The graph shows the relative TsAP protein expression levels determined by densitometry from three independent experiments. *P < 0.01 compared with ML and NBL group. **D** qPCR analysis of TsAP mRNA transcription level at diverse *T. spiralis* stages. The TsAP mRNA from ML, IIL, 3-day AW and NBL were isolated and amplified by qPCR. The TsAP transcription level was calculated according to the Ct (2^-ΔΔCt^) method. The fold change of TsAP genes normalized to GAPDH served as an internal control gene. Three repeats for each sample were performed. * *P* < 0.01 compared to muscle larvae stage.

The results of western blot showed that anti-rTsAP serum recognized the natural TsAP protein in soluble somatic proteins of diverse *T. spiralis* stages (ML, IIL, AW and NBL) (Figure 3A, B), indicating that TsAP was expressed at all *T. spiralis* lifecycle stages. The relative quantitative analysis revealed that the TsAP protein expression levels in IIL and AW were evidently higher than those in the other two stages (ML and NBL) (Figure 3C) (*P *< 0.01).

### qPCR analysis of TsAP mRNA transcription level at diverse stages

The qPCR results showed that the TsAP mRNA transcription was observed at four worm stages (ML, IIL, 3-day AW, and NBL) (Figure 3D). The difference of TsAP transcriptional level in four worm stages was significantly different (*F* = 110.851, *P *< 0.001). The TsAP mRNA transcription level in IIL and AW stages was obviously higher than those in the ML stage (3.03 and 1.72 fold, *P* < 0.01); whereas its transcription level in the NBL stage was lower than those in the ML stage (0.14 fold, *P* < 0.01). Additionally, The TsAP transcription level in IIL was also significantly higher than those in the AW stages (*F* = 32.042, *P* < 0.001).

### Recognition of native TsAP at various *T. spiralis* stage worms by IFT

**Figure 4:**
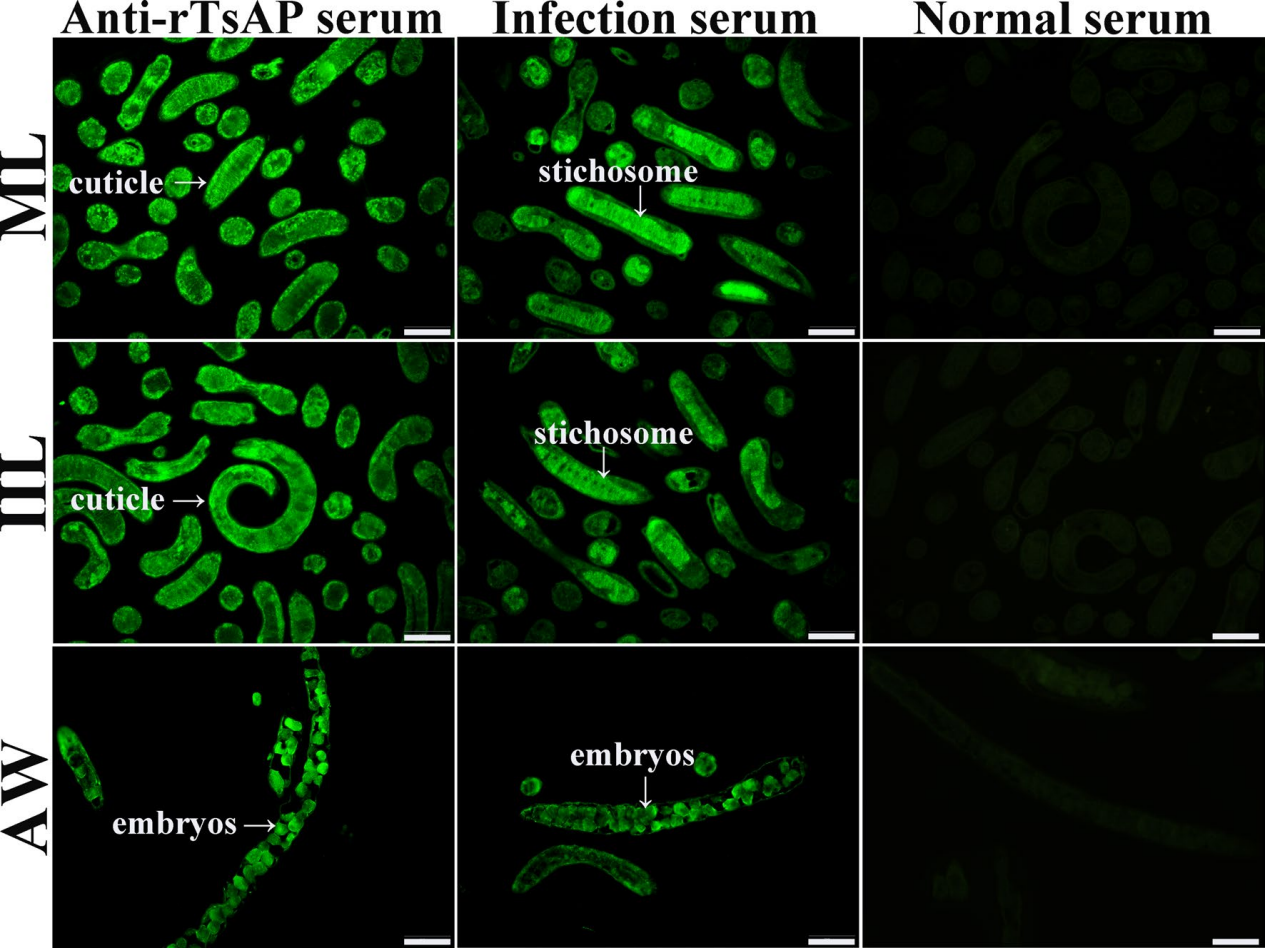
**Recognition and immunolocalization of TsAP in cross-sections of diverse*T. spiralis*stages by IFT using anti-rTsAP serum.** The worm sections were probed with anti-rTsAP serum, and immunostaining was identified at the cuticle of ML, IIL and AW, and female intrauterine embryos. But normal mouse sera did not identify any worm tissue components of the parasitic nematode. Scale-bar = 50 μm.

The IFT results showed that anti-rTsAP serum recognized the native TsAP on worm cross-sections, the immunostaining was principally located at the cuticle of ML, IIL and AW, and female intrauterine embryos (Figure 4). No worm tissue components of the parasitic nematode were identified in sera from normal mice.

### Enzymatic activity of rTsAP

**Figure 5:**
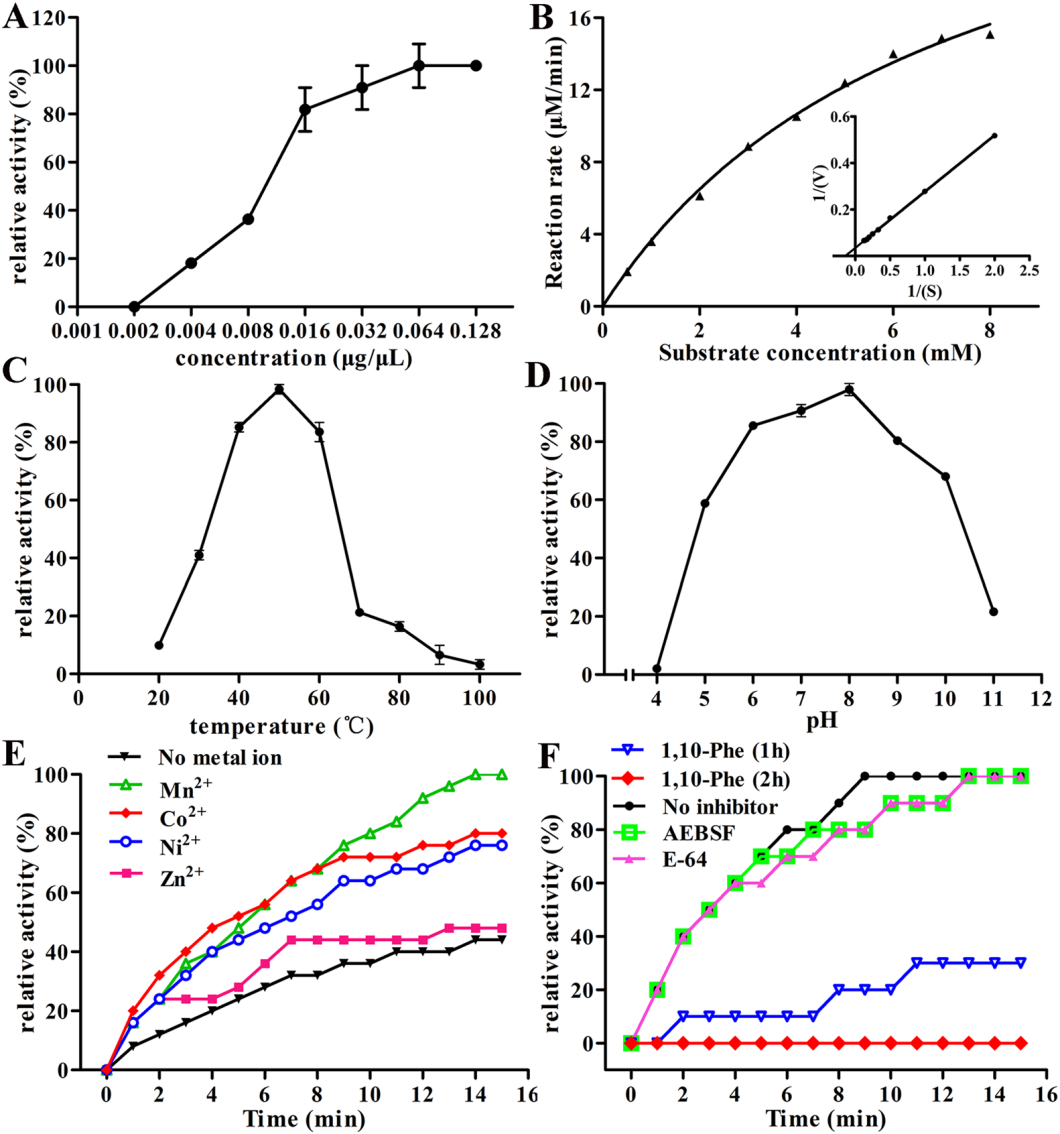
**Enzymatic activity of rTsAP. A** Enzyme activity of rTsAP at different concentrations. **B** Michaelis–Menten plot and Lineweaver–Burk plot. **C** rTsAP enzyme activity at different temperatures. **D** rTsAP enzyme activity at different pH values. **E** Effects of different metal ions on rTsAP enzyme activity. **F** Effects of different inhibitors on rTsAP enzyme activity.

The enzymatic activity of rTsAP gradually increased with increasing rTsAP concentration, and stabilized at a concentration of 0.064 μg/μL (Figure 5). The optimal reaction temperature was 50 °C, and the optimal pH was 8.0. The enzymatic activity of rTsAP was significantly enhanced by three metal ions (Mn^2+^, Co^2+^ and Ni^2+^), but not by Zn^2+^ ions, and the enhancement role was Mn^2+^ > Co^2+^ > Ni^2+^. The inhibitor 1,10-Phenanthroline has an inhibitory effect on rTsAP enzyme activity; the other two protease inhibitors (AEBSF and E-64) have no suppressive effect on rTsAP enzyme activity. The hydrolysis effect of rTsAP on the substrate Leu-pNA obeyed simple Michaelise-Menten kinetics, with kinetic parameters V_max_ of 28.99 μM min^−1^ and K_m_ of 14.04 mM.

### Binding of rTsAP and IEC proteins determined by ELISA

**Figure 6:**
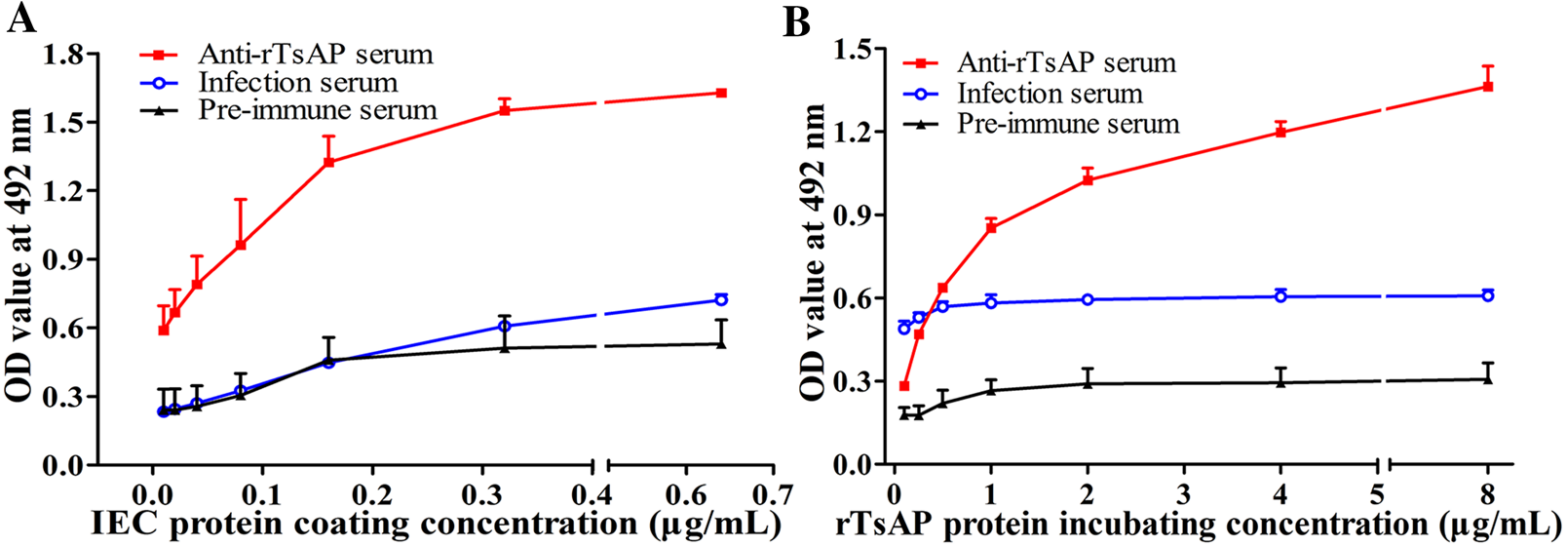
**Capacity of rTsAP to bind with IEC protein determined by ELISA. A** Binding between serial diluted coating IEC protein (0.01, 0.02, 0.04, 0.08, 0.16, 0.32 and 0.64 μg/mL) and 5 μg/mL rTsAP protein. **B:** Binding between 0.32 μg/mL coating IEC protein and serial diluted rTsAP protein (0.10, 0.25, 0.50, 1.00, 2.00, 4.00 and 8.00 μg/mL). The binding of rTsAP with IEC proteins is dose-dependent of rTsAP and IEC proteins.

The binding between rTsAP and IEC protein was measured by ELISA. The results revealed that there was a strong interaction of rTsAP with IEC proteins. The absorbance was dose dependent of IEC proteins (*r* = 0.878, *P *< 0.01) and showed an elevating trend along with the increasing IEC coating concentration (*F* = 127.553, *P* < 0.001) (Figure 6A). Furthermore, the absorbance was also rTsAP dose-dependent (*r *= 0.868, *P* < 0.05) and exhibited a positive trend with elevation of rTsAP concentrations (*F* = 938.272, *P *< 0.001) (Figure 6B).

### Binding of rTsAP and IEC proteins determined by Far western

**Figure 7:**
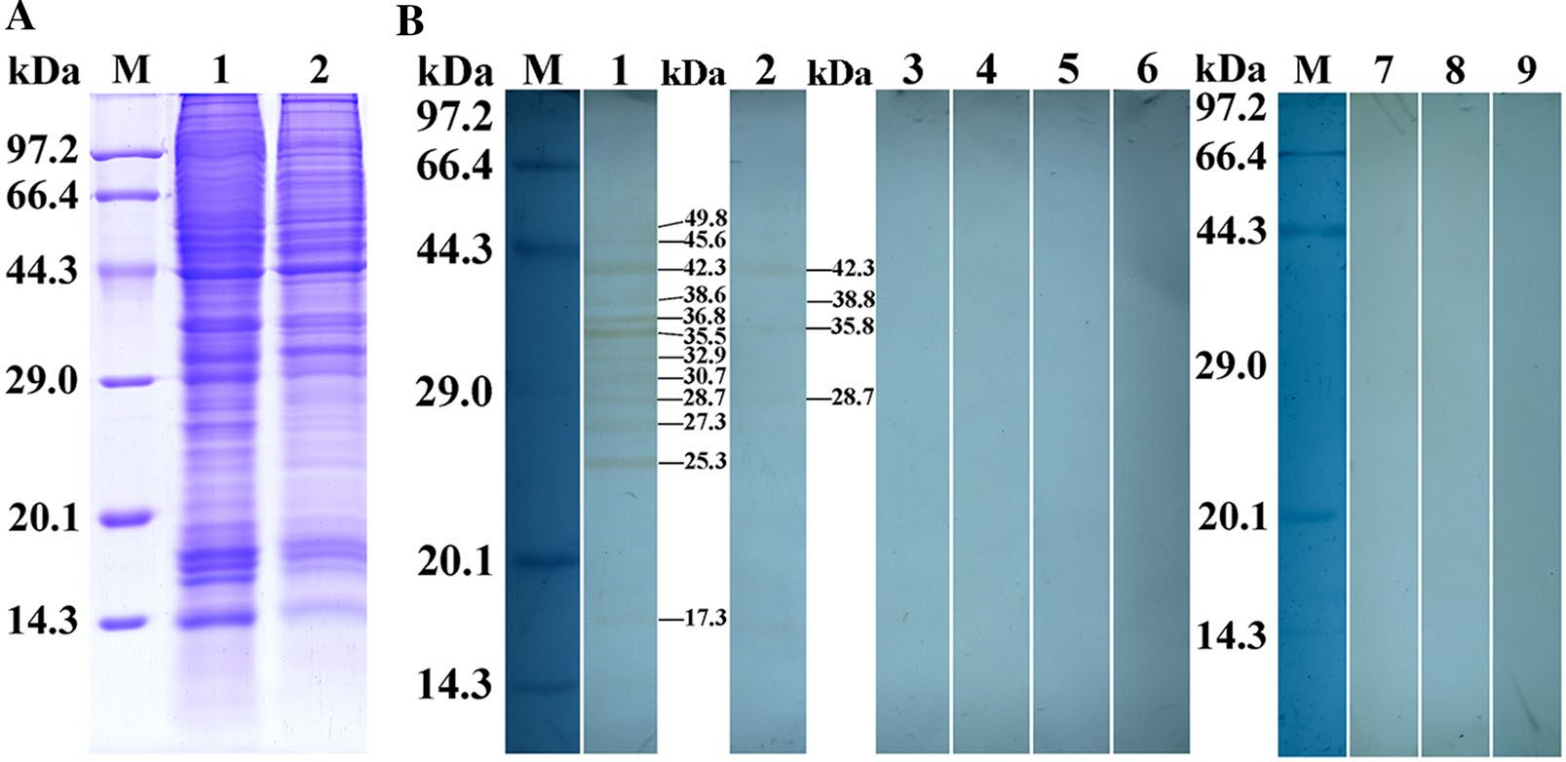
**Far-western blotting of rTsAP binding with IEC proteins. A** SDS-PAGE analysis of IEC (lane 1) and C2C12 (lane 2) lysates. **B** Specific binding between rTsAP and IEC proteins. The membrane with the IEC proteins (lanes 1–6) was incubated by rTsAP (lanes 1–3) or BSA (lanes 4–6). Specific binding bands of rTsAP with IEC were identified by anti-rTsAP serum (lane 1) and infection serum (lane 2), but not by preimmune serum (lane 3). No binding of BSA with IEC were detected by anti-rTsAP serum (lane 4), infection serum (lane 5) and preimmune serum (lane 6). No binding of rTsAP with C2C12 was observed using anti-rTsAP serum (lane 7), infection serum (lane 8) and preimmune serum (lane 9).

After IEC protein was incubated with rTsAP, about 12 protein bands of 17.3–49.8 kDa were recognized by anti-rTsAP serum, and 4 bands of 28.7–42.3 kDa were recognized by infection serum. No IEC proteins pre-incubated with rTsAP were identified by preimmune serum, and not any C2C12 components pre-incubated with rTsAP were detected using anti-rTsAP serum or infection serum (Figure 7). The results indicate that there is a specific binding between TsAP and IEC proteins.

### Binding of rTsAP and IEC and its cellular localization

**Figure 8:**
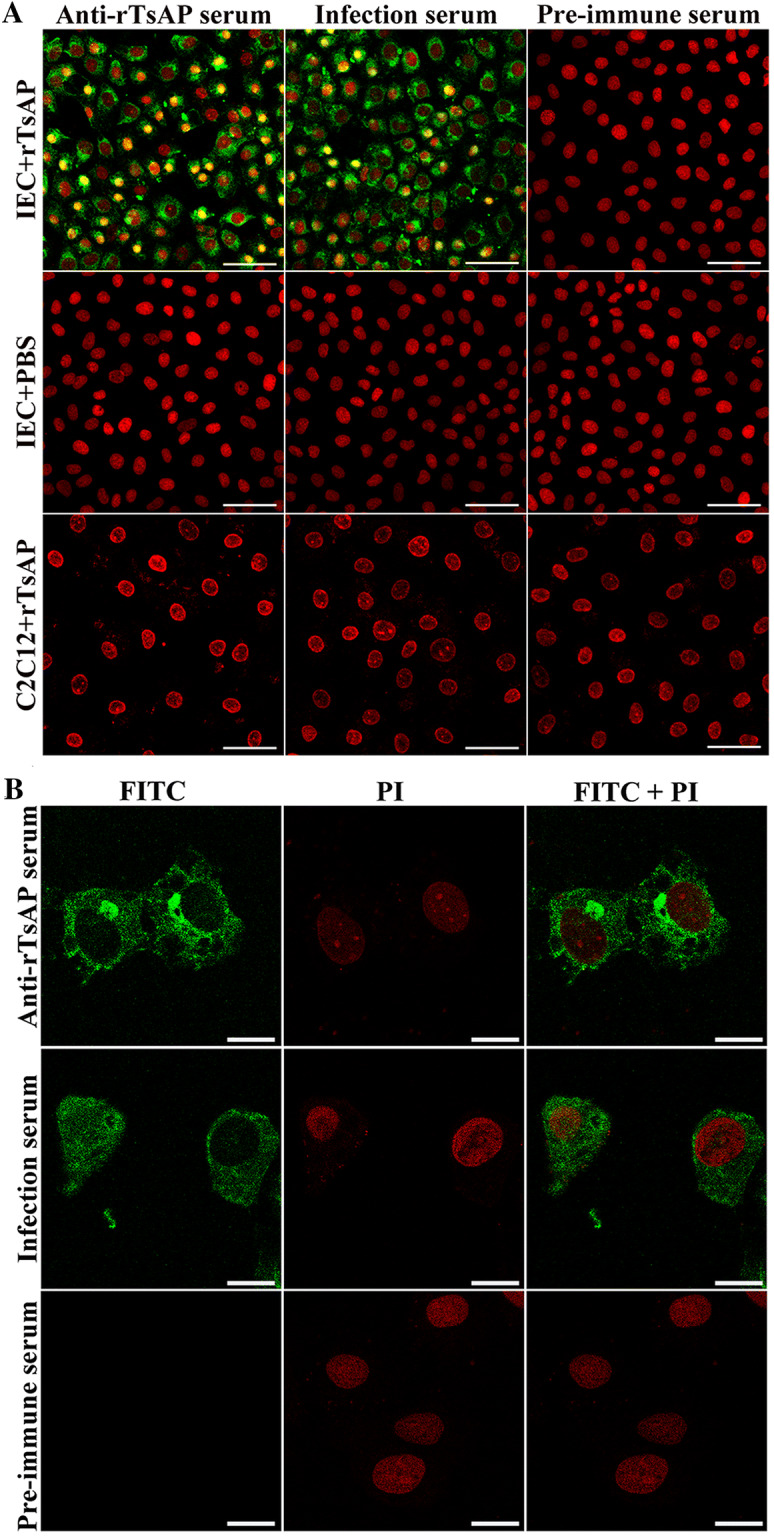
**Specific binding of rTsAP with IEC and cellular localization. A** IFT analysis of specific binding of rTsAP with IEC. The IEC or C2C12 cells were pre-incubated with rTsAP or PBS. After blocking and washes, the IEC and C2C12 were incubated with anti-rTsAP serum, infection serum or pre-immune serum, followed by the incubation of FITC-conjugated anti-mouse IgG. Cell nuclei were re-dyed red by propidium iodide (PI). Immunostaining was observed on the surface of IEC probed by anti-rTsAP serum and infection serum, but not by pre-immune serum. Scale-bars: 25 μm. **B** Cellular localization of rTsAP in IEC by confocal microscopy. The IEC were first pre-incubated with rTsAP, subsequently probed using anti-rTsAP serum, infection serum or pre-immune serum, finally stained using FITC-conjugated anti-mouse IgG. PI was utilized to dye cell nuclei red. Immunostaining was principally located within IEC cytoplasm. Scale-bars: 5 μm.

The IFT results showed that when the IEC were pre-incubated with rTsAP, green fluorescence was observed on the surface of IEC probed by anti-rTsAP serum and infection serum, but not by pre-immune serum. The IEC pre-incubated with PBS alone did not exhibit any immunostaining. No fluorescent staining on C2C12 was detected when the C2C12 pre-incubated with rTsAP was probed using anti-rTsAP serum or infection serum (Figure 8A). The results of confocal microscopy indicate that the immunostaining was principally located within IEC cytoplasm (Figure 8B), suggesting that there is a specific binding of rTsAP with IEC and the binding site is located mainly within the cytoplasm.

### rTsAP facilitation or anti-rTsAP serum suppression on larva penetration into IEC

**Figure 9:**
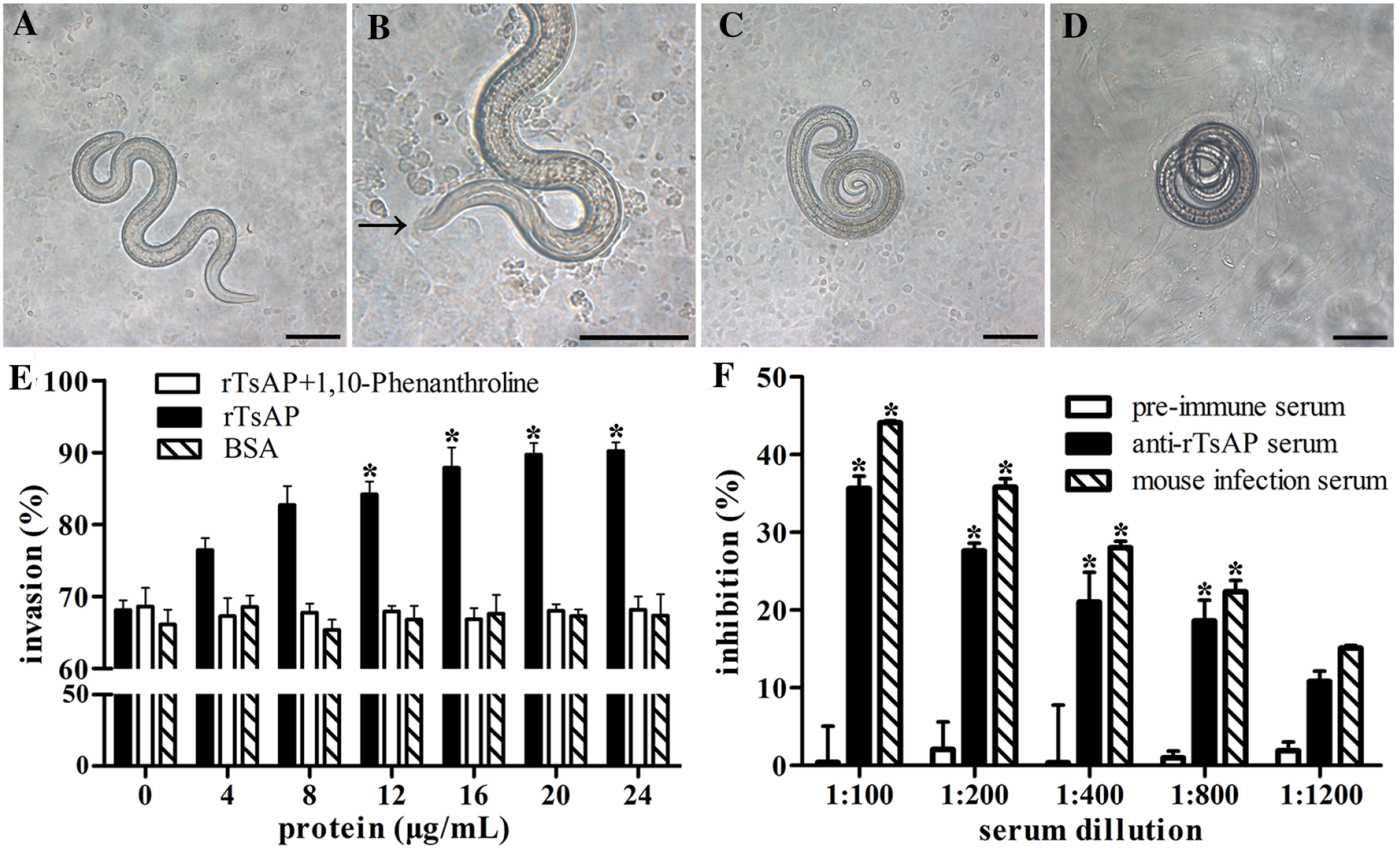
**rTsAP facilitation or anti-rTsAP serum suppression on IEC invasion by intestinal infective larvae (IIL).***Trichinella spiralis* ML were first activated into the IIL larvae with 5% swine bile for 2 h at 37 °C, the IIL were added onto the IEC monolayer and the penetration was examined under a microscope at 2 h after co-culture. **A**, **B** Larvae invaded into the IEC monolayer was active, and the integrity of the IEC monolayer invaded by the IIL larvae was destroyed. **C** Non-penetrated IIL coiled on the surface of the IEC monolayer. **D** Non-penetrated IIL coiled on the surface of the C2C12 monolayer. **E** rTsAP promoted the IIL invasion of IEC, but the promotion was suppressed by enzymatic inhibitor phenanthroline. **F** Inhibition of anti-rTsAP immune serum on the IIL invasion of IECs. * *P* < 0.01 compared to the BSA, rTsAP + phenanthroline or pre-immune serum group. Scale-bars: 100 μm.

After being co-cultivated with IEC monolayer for 2 h, the IIL penetrated into the IEC monolayer (Figure 9). When the medium was supplemented with rTsAP and co-cultivated with IIL larvae for 2 h, an evident facilitation role of rTsAP on parasite penetration into IEC was observed. This facilitation was rTsAP dose-dependent (*r *= 0.916, *P *< 0.001), and shows a rising trend with the increase of the rTsAP dose (*F *= 215.761, *P *< 0.001), but the rTsAP treated with aminopeptidase inhibitor (1, 10-Phenanthroline) and BSA did not promote the larval penetration. When serial dilutions of anti-rTsAP serum were replenished into the medium and co-cultured with IIL larvae for 2 h, anti-rTsAP serum (1:100–1:800) resulted in a significant suppression of larval penetration into IEC relative to the pre-immune serum group (*P *< 0.01). The suppressive role was dose-dependent of anti-rTsAP antibodies (*r *= 0.924, *P *< 0.001) and showed a reducing trend with the increasing serum dilutions (*F *= 175.096, *P *< 0.001). Nevertheless, pre-immune serum did not have any inhibitory effects on the larval penetration into enterocytes.

### Suppression of siRNA 842 on TsAP expression, activity and the in vitro intrusion

**Figure 10:**
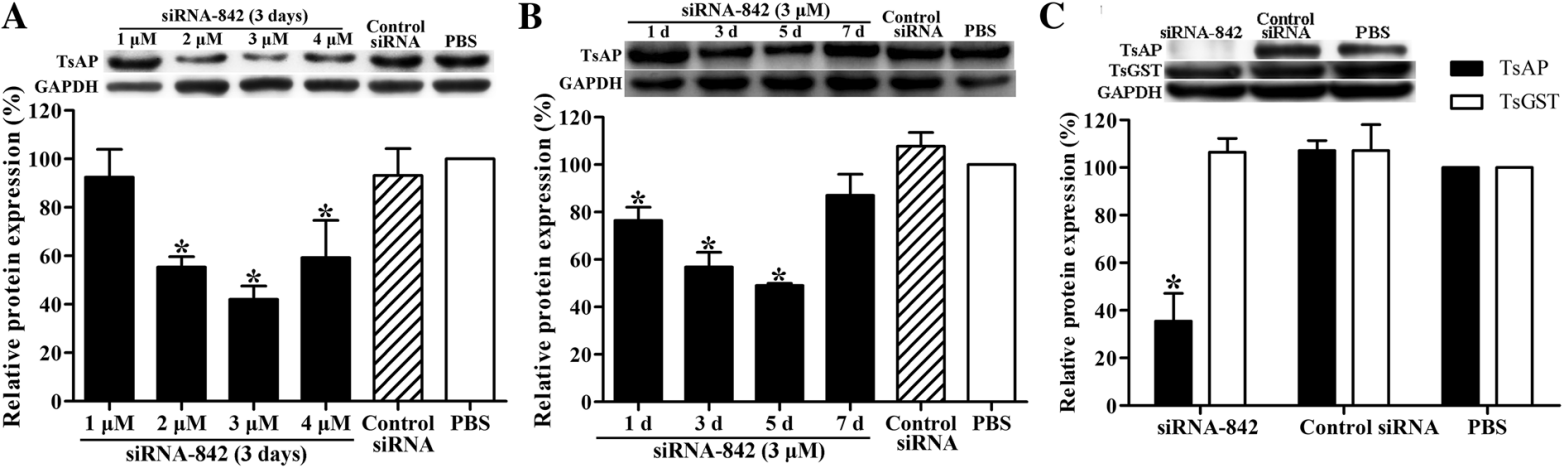
**Suppression of TsAP protein expression in siRNA 842-transfected ML on western blot analysis. A** TsAP expression in ML transfected with serial dilutions of siRNA 842. **B** TsAP expression in ML at 1–7 days after transfection with 3.0 μM siRNA 842. **C** Expression levels of TsAP and *T. spiralis* glutathione S-transferase (TsGST) in ML transfected with TsAP-specific siRNA 842.

When the ML were transfected with 2.0, 3.0 and 4.0 μM siRNA 842 and cultured for 3 d, TsAP protein expression level was suppressed by 44.64, 57.89 and 40.71%, respectively, compared to the PBS group (*P *< 0.01) (Figure 10A). At days 1, 3 and 5 after transfection with 3 μM siRNA 842, the TsAP protein expression level was inhibited by 23.59, 43.17 and 50.90% (*P *< 0.01) (Figure 10B). In ML treated with siRNA 842, the protein expression level of a *T. spiralis* glutathione S-transferase (TsGST) was not inhibited (*P *< 0.01) (Figure 10C), confirming that siRNA 842 is TsAP-specific.

**Figure 11:**
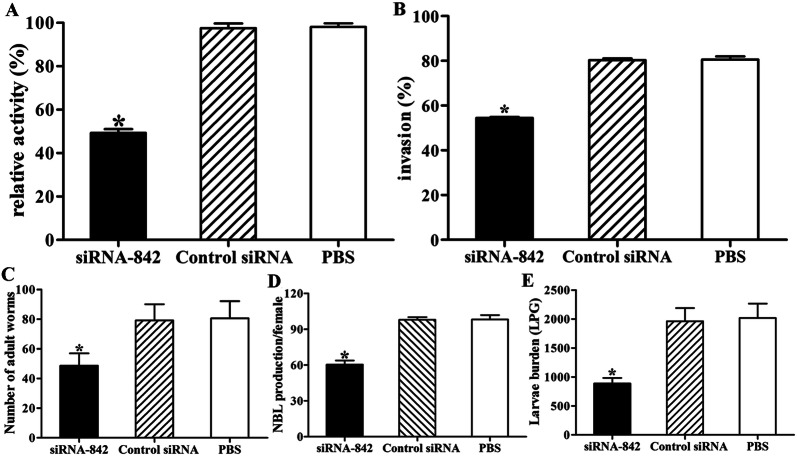
**Suppression of siRNA 842 on TsAP activity, larval invasion, intestinal adult burden and fecundity, and muscle larval burden. A** Suppression of siRNA 842 on TsAP enzymatic activity. The ML were transfected with 3 μM siRNA 842 and cultured for 3 days, the TsAP enzymatic activity in crude proteins of transfected ML to cleave the substrate Leu-pNA was assayed. **B** Suppression of siRNA 842 on larva intrusion of IEC. The siRNA 842-transfected ML were first activated into the IIL with swine bile for 2 h at 37 °C, subsequently the IIL larvae were inoculated onto the IEC monolayer and the intruded worms were examined under microscopy at 2 h after incubation. The results are expressed as the percent of penetrated larvae to all larvae utilized in each test and shown as mean ± SD of 3 independent tests. *P < 0.001 compared with PBS and control siRNA group. **C** Intestinal adult worm burden at 6 dpi. **D** Newborn larvae (NBL) production of adult females adults in 72 h. Worm burden (*n* = 10) and NBL production (*n* = 30) are shown as mean ± SD from siRNA 842, control siRNA and PBS group. **E** Muscle larvae per gram (LPG) of tissues at 60 dpi. *P < 0.001 relative to the PBS or control RNA group.

The TsAP enzymatic activity in crude proteins of ML transfected with 3 μM siRNA 842 for 3 days was assayed. The results show that the TsAP enzyme activity of siRNA 842 treated ML to hydrolyze the substrate Leu-pNA was reduced by 49.72% relative to non-treated ML (*F *= 1246.154, *P* < 0.001) (Figure 11A). The aggressive capacity of the bile-activated IIL from siRNA 842-transfected ML was inhibited by 32.35% (*F *= 921.579, *P* < 0.001) (Figure 11B). However, TsAP enzymatic activity and worm aggressive capacity were not inhibited when the parasites were transfected by control siRNA.

### Suppression of siRNA 842 on the in vivo larval intrusion, development and fecundity

The results of the in vivo larval intrusion revealed that mice challenged with siRNA 842 transfected ML showed a 39.58% reduction of intestinal AW and a 56.03% reduction of ML relative to the PBS group (Figure 11C, E) (*F*_AW_ = 50.033, *F*_ML_ = 180.217; *P* < 0.001). However, the mice infected with the ML transfected by control siRNA did not show any significant reduction of AW and ML burdens (*F*_AW_ = 0.077, *F*_ML_ = 0.283; *P *> 0.05).The production of NBL deposited by each female from the siRNA 842 group was distinctly inferior to those from the PBS group (Figure 11D) (*F *= 171.278,*P *< 0.001).

**Figure 12:**
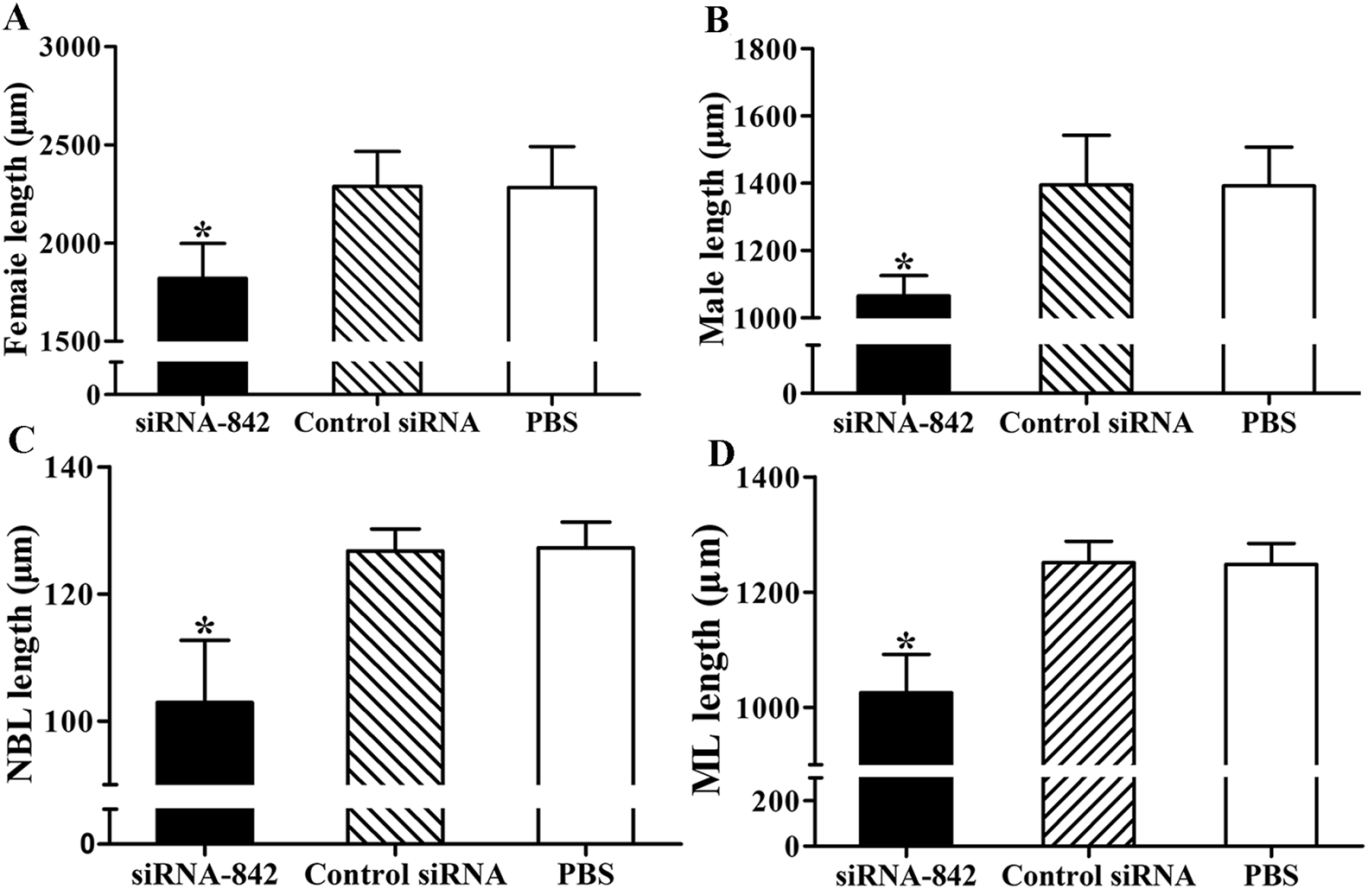
**The lengths of*T. spiralis*females (A), males (B), NBL (C) and ML (D) collected from mice challenged with muscle larvae transfected with siRNA 842, control siRNA or PBS (*n* = 15).** *P < 0.001 in comparison with the control siRNA and PBS group.

Moreover, the length of male and female AW recovered from the siRNA 842 group was prominently shorter than those from PBS groups (Additional file 2; Figure 12) (*F*_male_ = 94.371, *F*_female_= 43.018, *P *< 0.001). Additionally, the length of the NBL and ML from the siRNA 842 group were also significantly shorter than those from the PBS group (*F*_NBL_= 78.891, *F*_ML_ = 129.492; *P *< 0.001). But, the differences in the length of the AW, NBL and ML collected from control siRNA and the PBS group had no statistical significance (*P *> 0.05). The results indicate that silencing of the TsAP gene inhibited larval infectivity, developmental capacity, and as a result, reduced the adult burden and female fecundity, and alleviated the *T. spiralis* infection in challenged mice with siRNA 842 transfected ML.

## Discussion

Aminopeptidase is a kind of exopeptidase that catalyzes the sequential removal of amino acids from the N terminus of peptides and proteins. The aminopeptidase belongs to the Peptidase M17 family, which can also be grouped into diverse aminopeptidases on the basis of its main substrate. Leucine aminopeptidase (LAP) is one representative group of them [16]. In parasites, aminopeptidase might play significant biological roles such as molting, surface membrane remodeling, egg hatching and digestion for the parasite survival in hosts [62]. Silencing of the LAP gene using RNAi blocks the hatching of *Schistosoma mansoni* eggs [63]. In the bovine filaria *Setaria cervi*, this enzyme participated in parasite feeding, digestion, cuticle remodeling, egg hatching and embryogenesis [64]. Recent studies showed that *Plasmodium falciparum* aminopeptidase 1 (PfA-M1) is involved in the hemoglobin digestion cascade that provides most of the amino acids necessary to the parasite’s metabolism, it is regarded as a promising new therapeutic target [65]. The mutation of *Caenorhabditis elegans* aminopeptidase 1 (ANP-1) induced small body size in the L4/young adult stage of *C. elegans.* Furthermore, deletion of ANP-1 resulted in a shortened lifespan and fewer egg layings, indicating that ANP-1 participates in development and aging of *C. elegans* [66]. But there are no reports of biochemical and functional analysis of TsAP in the literature.

In order to obtain TsAP with enzymatic activity and to ascertain its biological roles in the *T. spiralis* life cycle, the complete TsAP sequence was cloned into pQE-80L plasmid and expressed in *E. coli* in this work. Sequence analysis shows that TsAP had an identity of 99.40, 99.21, 98.61 and 98.61% with aminopeptidase of the 4 encapsulated *Trichinella* species (*T. murrelli*, *T. britovi*, *T. nativa* and *T. nelsoni*). A phylogenetic tree shows the monophyletic group of 11 species/gene types of the genus *Trichinella.* On the basis of the aminopeptidase phylogenetic analysis, *T. spiralis* has a closer evolutionary relationship with 7 encapsulated species of the genus *Trichinella* as shown in Figure 1C. The SMART analysis shows that TsAP carried two functional domains of Peptidase-_ M17 which is a catalyser of the hydrolysis of N-terminal amino acid residues. It is suggested that TsAP is a metallo-aminopeptidase belonging to the M17 family of proteases. TsAP might be involved in degradation of the hydrolyzed peptide fragments of the host’s intestinal epithelial components and participates in the larval invasion of the IEC. It is likely related with molting, cuticle remodeling and fecundity, and plays a role in important biological functions in *T. spiralis* larval development in the host [17, 63]. After being purified, the rTsAP was strongly immunogenic and served to generate anti-rTsAP antibodies. Immunization of mice with rTsAP elicited high levels of specific anti-rTsAP antibody IgG response. qPCR shows that TsAP transcription was observed at diverse *T. spiralis* phase worms (ML, IIL, AW and NBL), and the TsAP transcription level in the IIL stage was significantly higher than those in the other three worm stages. On western blot analysis, rTsAP was recognized by anti-rTsAP serum and infection serum. And the native TsAP in crude somatic proteins of four *T. spiralis* lifecycle stages (ML, IIL, AW and NBL) was identified by anti-rTsAP serum, indicating that TsAP was expressed at all *T. spiralis* life stages. The relative quantitative analysis revealed that the TsAP protein expression level in IIL and the adult stage were evidently higher than those in ML and NBL stages. The IFT results demonstrate that native TsAP was principally located at the cuticle of this parasitic nematode, suggesting that TsAP is an essential interaction protein between parasite surface and the host. The high levels of TsAP mRNA and protein expression in the IIL stage suggest that TsAP might be an invasion-related protein and might play an important role in parasite invasion and eliciting early immune response [38, 67].

The results of the enzymatic activity assay showed that rTsAP has the enzymatic activity of natural aminopeptidase to hydrolyze the substrate Leu-pNA with an optimal temperature of 50 °C and optimal pH of 8.0. The rTsAP enzymatic activity was significantly enhanced by three metal ions (Mn^2+^, Co^2+^ and Ni^2+^), indicating that TsAP is a leucyl-aminopeptidase that requires divalent metal cations and a basic optimal pH for catalysis [45]. A biochemical characterization indicates that the purified rTsAP is suitable for a biological functional assay. The protein interaction between rTsAP and IEC proteins was evaluated in this work. The results of ELISA and Far western revealed that there is a strong specific binding between rTsAP and IEC proteins, and this protein binding is dose-dependent of rTsAP and IEC proteins. The IFT and confocal microscopy verified that there is a specific binding of rTsAP with IEC and the binding site is located mainly within the IEC cytoplasm. Previous studies indicate that when the IIL larvae were co-cultivated with the IEC monolayer, the larvae produced several proteases that entered into the IEC [12].

The in vitro larval penetration experiment indicates that rTsAP obviously accelerated the parasite penetration into IEC, whereas anti-rTsAP serum evidently inhibited the worm penetration, and this acceleration or inhibition was dose-dependently related with rTsAP or anti-rTsAP antibodies. Moreover, the promotion role of rTsAP on larval invasion could be significantly inhibited by the inhibitor (1,10-Phenanthroline). The penetration acceleration might be related with the specific binding of TsAP and IEC [26, 51]. The inhibitory role of anti-rTsAP antibodies on the worm penetration of IEC might be due to the formation of a cap-like immune complex of TsAP and anti-TsAP antibodies at the worm anterior, which interrupted the direct contact between the parasite and enterocytes, and as a result, suppressed worm penetration [68]. When *T. spiralis*-infected murine sera were used in the in vitro penetration, their inhibitory effect on the penetration was higher than those of anti-rTsAP serum. This is likely because specific antibodies against other *T. spiralis* invasion-related proteins (e.g., nudix hydrolase, serine protease, serine protease inhibitor, etc.) in infection sera also participated in the inhibitory role on the invasion [39, 52, 56]. Nevertheless, it is necessary to identify and characterize the IEC proteins that interact with TsAP by means of the yeast two-hybrid, co-immunoprecipitation and mass spectrometry in future studies.

In parasitic nematodes, RNA interference is the current main tool for functional analysis of genes. RNAi technique has been used to investigate the functions of some *T. spiralis* genes [37]. In order to testify the physiological role of TsAP in the parasite invasion, development and reproduction, the TsAP-specific siRNA 842 was transfected into the ML with an electroporation method in this work. The results show that when the ML were transfected with 3 μM siRNA 842, the TsAP expression level after transfection was reduced by 50.90%. The TsAP enzyme activity of siRNA 842 treated ML was suppressed by 49.72%. Meanwhile, the invasive capacity of the bile-activated IIL from siRNA 842-transfected ML was inhibited by 32.35%. The results demonstrate that silencing of the TsAP gene by siRNA mediated RNAi obviously reduced the expression levels of TsAP protein and its enzymatic activity in *T. spiralis* larvae, and significantly inhibited the invasive capacity of the larvae to penetrate into the host’s IEC. The results also verified that TsAP participates in *T. spiralis* invasion of the intestinal epithelium. Furthermore, the results of the in vivo challenge experiment revealed a 39.58% reduction of intestinal AW at 6 dpi after the mice were challenged with siRNA-842-transfected *T. spiralis* ML. The intestinal worm development and female reproductive capacity were also significantly suppressed by TsAP-specific siRNA, as demonstrated by shorter adults and lower female fecundity compared to control siRNA and PBS groups. Other studies showed that deletion of thr TgAP gene in *Toxoplasma gondii* through a CRISPR/Cas9 knockout system inhibits the attachment/invasion, and growth of this protozoon [69]. Knockdown of aminopeptidase gene by RNAi resulted in dramatic reductions of *Caenorhabditis elegans* fecundity [70]. These findings demonstrated that TsAP plays a crucial role for the invasion, development and fecundity of *T. spiralis*.

In conclusion, TsAP was transcribed and expressed highly at IIL and adult stages of *T. spiralis* lifecycle. It was principally located at the cuticle and the intrauterine embryos of this nematode. rTsAP has a strong immunogenicity and enzymatic activity of a natural aminopeptidase. rTsAP has the capacity to specifically bind to IEC in the IEC cytoplasm. rTsAP promoted larval penetration into IEC, whereas anti-rTsAP antibody suppressed larval intrusion; the promotion and suppression were dose-dependently related to the rTsAP or anti-rTsAP antibodies. Silencing of the TsAP gene by specific siRNA obviously reduced the levels of TsAP expression and enzymatic activity. RNAi also inhibited the worm invasive capacity, development and fecundity. These results indicate that TsAP participates in the invasion, development and fecundity of *T. spiralis* and it might be a candidate target for anti-*Trichinella* vaccines.

## Supplementary information

**Additional file 1. Sequence alignment of*Trichinella spiralis*aminopeptidase gene (EFV57052) with other*Trichinella*species or genotypes.** Clustal X and BOXSHADE were used to analyze the sequences, distinct differences were observed in various *Trichinella* species/genotypes. Black shades indicate that residues identical to TsAP, and grey shades show the conservative substitutions.

**Additional file 2. Morphology of*T. spiralis*adult, NBL and ML collected from mice challenged with muscle larvae transfected with siRNA 842, control siRNA or PBS.** Scale bar = 100 μm.

## Abbreviations

AW: Adult worms
IECs: Intestinal epithelial cells
IFT: Immunofluorescence test
IIL: Intestine infectious larvae
LAP: Leucine aminopeptidase
ML: Muscle larvae
MW: Molecular weight
NBL: Newborn larvae
OPD: *o*-Phenylenediamine dihydrochloride
pI: Isoelectric point
TsAP: *T. spiralis* aminopeptidase
TsGST: *T. spiralis* glutathione S-transferase
