# Supplementary figures and images for: Characterization of a Trichinella spiralis aminopeptidase and its participation in invasion, development and fecundity
Source: Vet Res. 2020 Jun 15;51:78. doi: 10.1186/s13567-020-00805-w (PMC7296678; doi:10.1186/s13567-020-00805-w)

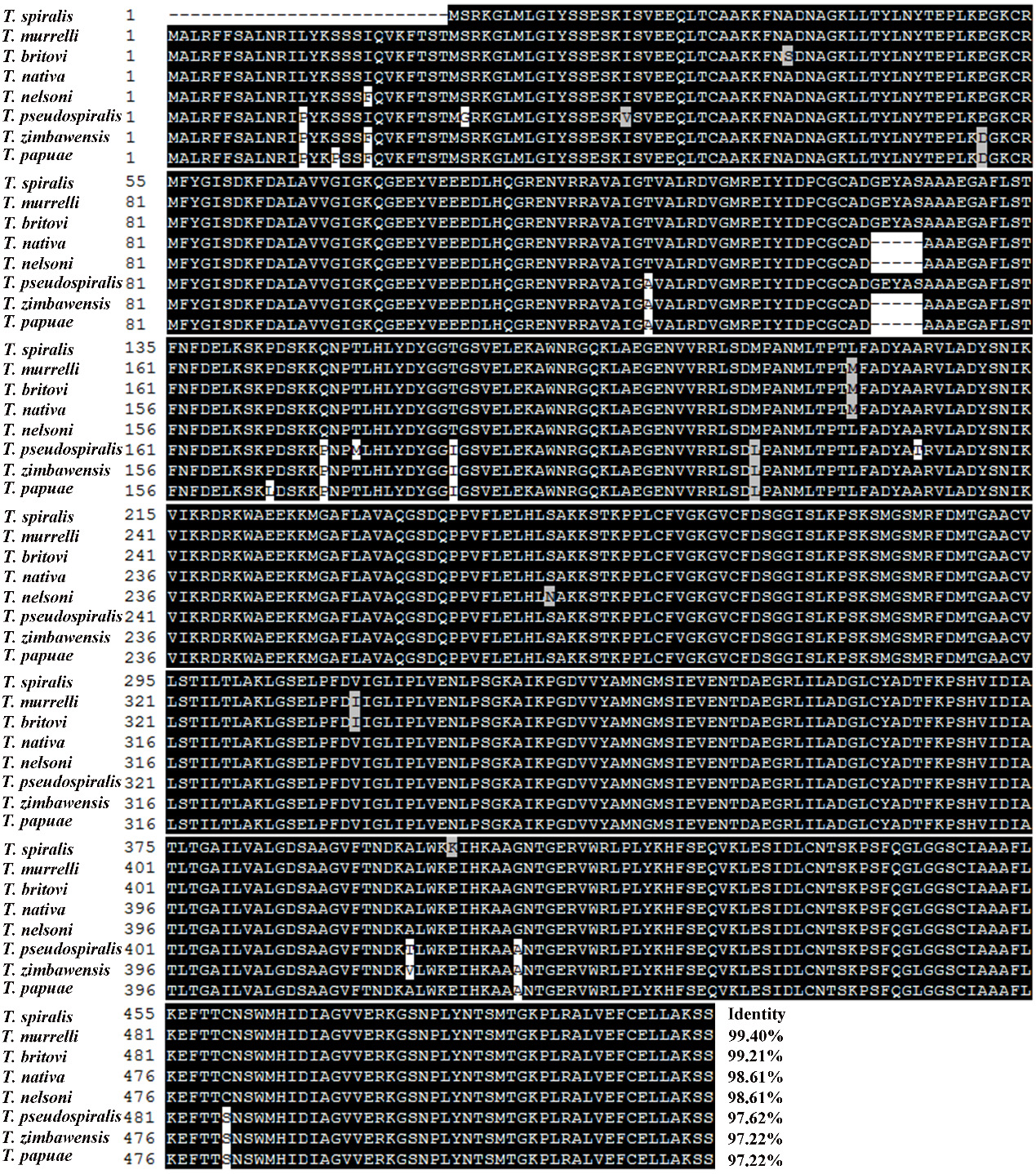

Supplement: Supplementary file 1 — Additional file 1. Sequence alignment ofTrichinella spiralisaminopeptidase gene (EFV57052) with otherTrichinellaspecies or genotypes. Clustal X and BOXSHADE were used to analyze the sequences, distinct differences were observed in various Trichinella species/genotypes. Black shades indicate that residues identical to TsAP, and grey shades show the conservative substitutions. [file 13567_2020_805_MOESM1_ESM.docx]

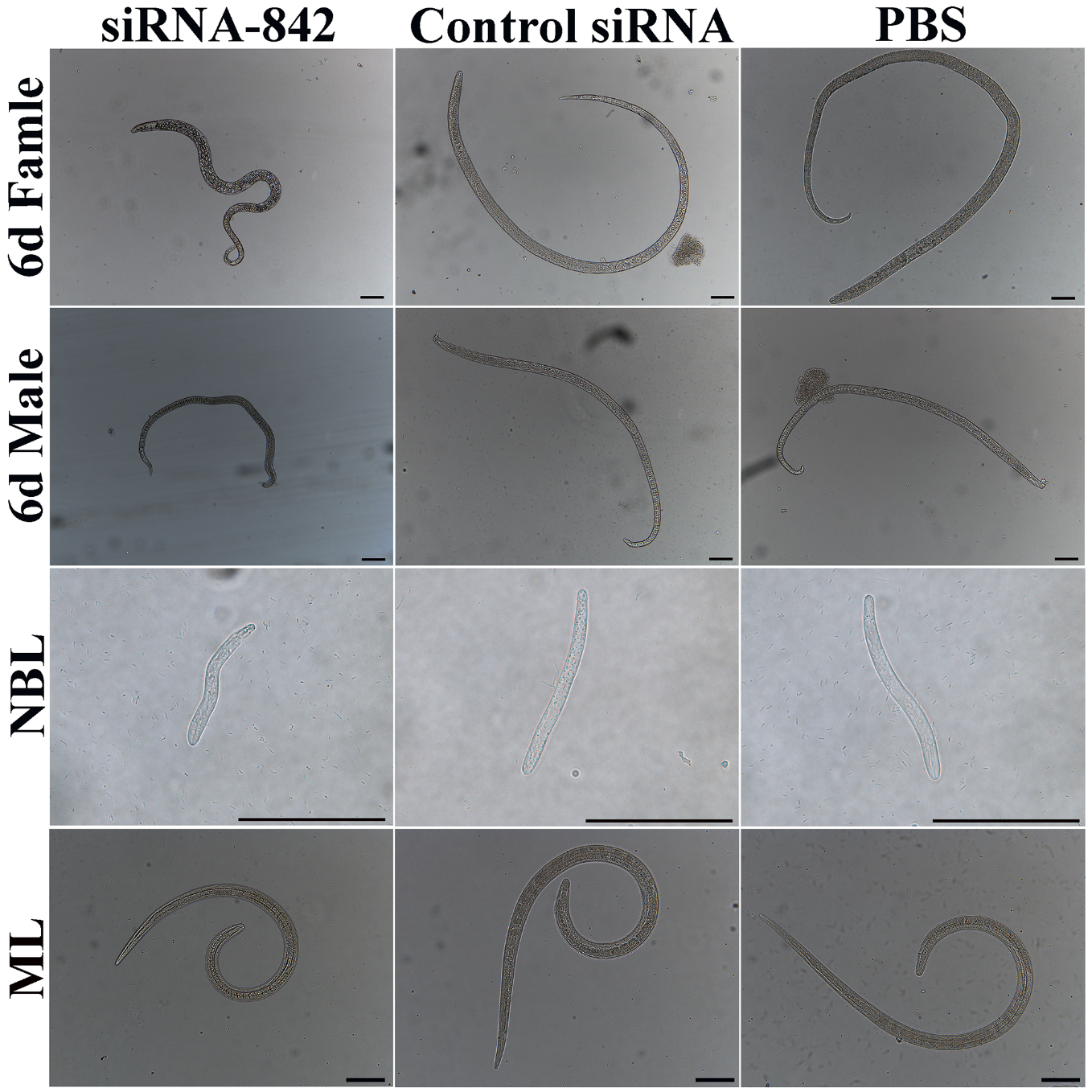

Supplement: Supplementary file 2 — Additional file 2. Morphology ofT. spiralisadult, NBL and ML collected from mice challenged with muscle larvae transfected with siRNA 842, control siRNA or PBS. Scale bar = 100 μm. [file 13567_2020_805_MOESM2_ESM.docx]
